# Use of design of experiments to optimize the production of microbial probiotic biofilms

**DOI:** 10.7717/peerj.4826

**Published:** 2018-07-10

**Authors:** Barbara Speranza, Arcangelo Liso, Maria Rosaria Corbo

**Affiliations:** 1 Department of the Science of Agriculture, Food and Environment (SAFE), University of Foggia, Foggia, Italy; 2 Department of Medical and Surgical Sciences, University of Foggia, Polo Biomedico, Foggia, Italy

**Keywords:** Biofilm, Probiotic, Bifidobacteria, Lactobacilli, Positive biofilms

## Abstract

Here, we describe the production of a probiotic biofilm through three intermediate steps: (1) measurement of the adhesion capacity of 15 probiotic strains to evaluate their tendency to form biofilm on different surfaces (stainless steel, glass, and polycarbonate); (2) evaluation of the effects of pH, temperature, cellular growth phase, agitation, and presence of surfactants on probiotic biofilm formation (BF) through the Design of Experiments (DoE) approach; (3) study of the effects of pH, temperature and surfactants concentration on probiotic BF using the Central Composite Design. Finally, we show that biofilms pre-formed by selected probiotics can delay the growth of pathogens, such as *Listeria monocytogenes* chosen as model organism. Among the tested strains, *Bifidobacterium infantis* DSM20088 and *Lactobacillus reuteri* DSM20016 were found to be as the probiotics able to ensure the greatest adhesion (over 6 Log CFU cm^2^) to the surfaces tested in a very short time (<24 h). Cellular growth phase and agitation of the medium were factors not affecting BF, pH exerted a very bland effect and a greater tendency to adhesion was observed when the temperature was about 30 °C. The results obtained in the last experimental phase suggest that our probiotic biofilms can be used as an efficient mean to delay the growth of *L. monocytogenes*: the λ phase length, in fact, was longer in samples containing probiotic biofilms (0.30–1.02 h) against 0.08 h observed in the control samples. A reduction of the maximum cell load was also observed (6.99–7.06 Log CFU mL^−1^ against about 8 Log CFU mL^−1^ observed in the control samples).

## Introduction

The capability of bacteria to form biofilms on biotic and abiotic surfaces has certainly the potential to generate critical problems; in fact, it is well documented that many pathogenic and spoilage microorganisms easily form biofilms on food-contact surfaces leading to serious hygienic risks (e.g. potential microbial contamination in food plants, lower shelf life of food products, potential transmission of diseases) and causing serious economic losses (failures in water systems, cooling towers, heat exchangers, etc.) (Speranza & Corbo, 2017).

However, not all biofilms cause problems and there are clear examples of their positive use, even if this aspect is rather neglected. For example, the potential of biofilm communities for bioremediation processes is actually used to treat toxic effluents and clean-up environmental pollutants and slow-degrading compounds. Other positive applications include the (a) treatment of industrial and municipal wastewaters, (b) application for in situ soil fertilization via N_2_ fixation, (c) reduction of ammonia and nitrate concentrations in aquaculture effluents, (d) treatment of sulphide-containing waste streams; and (e) production of biochemicals, comprising medicines, food additives or chemical additives for cleaning products, and antimicrobial compounds (Corbo, Speranza & Sinigaglia, 2009). In the context of positive applications, another potential use of biofilms to improve food safety was suggested: useful application of biofilms formed by lactic acid bacteria (LAB) against foodborne pathogens. To control growth of pathogens, food products have been packed with film activated by the addition of bacteriocins and/or other LAB-produced antimicrobial compounds (Iseppi et al., 2007; Mauriello et al., 2004); however, since these extracted substances could lose their activity over time, the direct use of the producer microorganism entrapped in biofilms has been proposed as a good solution to limit pathogens growth in/on foods (Guerrieri et al., 2009; Guillier et al., 2008; Speranza, Sinigaglia & Corbo, 2009). Importantly, the maintenance of a continuous metabolism could ensure an uninterrupted and stronger activity of the active substances, those being in loco produced. These antimicrobial agents such as organic acids, hydrogen peroxide, carbon dioxide, diacetyl, low molecular weight antimicrobial substances, bacteriocins and biosurfactants are also produced by probiotic bacteria which are known to exert a positive effect on the maintenance of human health. In particular, probiotic lactobacilli have long been known for their antimicrobial activity due to the release of biosurfactants; these compounds were generally produced, extracted and pre-adsorbed to surfaces to inhibit microbial growth or adhesion by pathogens (*Listeria monocytogenes*, *Salmonella arizonae*, *Escherichia coli*, and *Staphylococcus aureus*) (Gudiña, Teixeira & Rodrigues, 2010; Jones & Versalovic, 2009; Rodrigues et al., 2004, 2006a, 2006b, 2006c, 2006d, 2007) or yeasts (Fracchia et al., 2010). Notably, all the studies on this topic so far have proposed the use of biosurfactants and other compounds produced in greater quantities by lactobacilli when growing in a sessile form, but no study has explored yet the direct use of producer strains grown as biofilms.

In fact, very few studies have yet been conducted on the ability of probiotic microorganisms to form biofilm (especially belonging to the genus *Bifidobacterium*) even if it is recognized and documented their ability to colonize the gastrointestinal tract, indeed as biofilm. Taking advantage of the in vivo metabolism of sessile probiotic strains, a probiotic biofilm could be a useful mean to control the growth of pathogenic and spoilage bacteria standing up as an innovative biotechnological solution for industrial and medical applications. Recently, probiotics have shown a good effectiveness against several gastrointestinal disorders (Alfaleh et al., 2011; Chapman, Plosker & Figgitt, 2006; Guandalini et al., 2000; Johnston et al., 2011; Marseglia et al., 2007; Podolsky, 2012; Wall et al., 2011). Importantly, the potential application of probiotics was recently widened to prevent and treat different disease conditions, including oral, genitourinary and gynaecological problems (Alexandre et al., 2014; Bizzini et al., 2012; Mastromarino, Vitali & Mosca, 2013; Shida & Nomoto, 2013; Serban, 2014; Saha et al., 2012).

Taking into account these consideration, a probiotic biofilm formed ad hoc on medical devices (catheters, implants, braces, bite blocks or condoms) and on bathrooms’ surfaces (sink, bidet, toilet bowl, water closet or piece of furniture) could be a potential new tool against colonizing strains, since these surfaces are often implicated in nosocomial infections.

Thus, the present study aims at exploring the possibility to generate a specific probiotic biofilm; this main topic was addressed through three intermediate steps:

- Measurement of the adhesion capacity of 15 strains with probiotic potential in model systems and optimal growth conditions to evaluate their tendency to form biofilm on different surfaces (stainless steel, glass, and polycarbonate);
- Evaluation of the effects of pH, temperature, cellular growth phase, agitation, and presence of surfactants on probiotic biofilm formation (BF) to highlight the most significant variables;
- Study of the effects of pH, temperature and surfactants concentration on probiotic BF testing both an anionic surfactant and a non-ionic surfactant.

Finally, to test whether biofilm pre-formed by probiotics could delay the growth of pathogens, we conducted experiments evaluating their effect on *L. monocytogenes* growth chosen as model organism, due to its wide distribution in nature and importance both in food processing and medical environment.

## Materials and Methods

### Phase I: measurement of the adhesion capacity of 15 strains with probiotic potential

#### Bacterial strains and culture conditions

The probiotic strains used for this study are reported in Table 1. The bacterial strains were stored at −20 °C in MRS broth (Oxoid, Milan, Italy), whereas the yeasts were stocked on Sabouraud dextrose agar (SAB; Oxoid, Milan, Italy) slants at 4 °C. Before each assay, the bacterial and yeast strains were grown in their optimal media (OM), at their optimal conditions (see Table 1), until late exponential phase was attained. Cells cultures were successively harvested by centrifugation for 10 min at 4,500 rpm (4 °C) and the pellets were washed twice with sterile isotonic solution temperate at 4 °C and finally resuspended in physiological solution (0.9% NaCl) at a cell concentration of 1 × 10^8^ CFU mL^−1^. These cell suspensions were diluted in order to make a cell concentration of 10^3^ CFU mL^−1^ (working culture) for adhesion experiments. To guarantee reproducibility in the inocula preparation, the cell counts were standardized through the direct plate count method (Harrigan, 1998).

**Table 1:** Probiotic strains used in the study with the indication of their optimal media and growth conditions adopted.

Strains	Optimal Media (OM) and growth conditions
**Bifidobacteria**	
*B. animalis* DSM10140	MRS broth (Oxoid, Milan, Italy), added with cysteine 0.05% (w v^−1^) (Sigma-Aldrich, Milan, Italy) incubated at 37 °C for 24–48 h under anaerobic conditions
*B. subtilis* DSM20096	
*B. infantis* DSM20088	
*B. longum* DSM20219	
*B. breve* DSM20213	
**Lactobacilli**	
*L. plantarum* DSM2601	MRS broth (Oxoid, Milan, Italy) incubated at 30 °C for 24–48 h under anaerobic conditions
*L. casei* DSM20011	
*L.delbrueckii* DSM20081	
*L. paracasei* DSM20207	
*L. reuteri* DSM20016	
**Yeasts**	
*Kluyveromyces lactis* ATCC8585	Yeast extract peptone dextrose (YPD; Oxoid, Milan, Italy) incubated at 25 °C for 48 h
*S. cerevisiae boulardii* ATCCMYA-796	
S. cerevisiae W21*	
S. cerevisiae W40*	
S. cerevisiae W45*	

**Note:**

* Autochthonous yeasts isolated from wine (Petruzzi et al., 2014).

#### Slides preparation

Stainless steel (AISI-316, finish#2B; ARVEL, Naples, Italy), glass and polycarbonate resin (Lexan; Fedele s.r.l., Rome, Italy) were the surfaces chosen for the adhesion experiments. Before each experiment, the chips (2.5 × 5.0 × 0.05 cm) were opportunely treated as described by Speranza, Corbo & Sinigaglia (2011) and autoclaved at 121 °C for 15 min prior to use.

#### Biofilm formation assays

To promote BF, sterile chips were placed vertically into jars containing 45 mL of sterile OM and aliquots of the working culture were inoculated at 10^2^ CFU mL^−1^. All flasks were incubated at optimal temperatures (30 °C for lactobacilli, 37 °C for bifidobacteria, 25 °C for yeasts), under static conditions, for three days.

Biofilm cells were enumerated at 0.5, 1, 2, and 3 days after inoculum. At these times, chips were aseptically removed, rinsed with sterile distilled water, transferred into test tubes containing 45 mL of sterile saline and treated with a 20 Hz “Vibra Cell” sonicator (SONICS, Newcastle, CT, USA) for 3 min to detach sessile cells. Viable and cultivable cells were enumerated by serial dilutions in 0.9% NaCl solution and plating on OM. Results were expressed as Log CFU cm^−2^.

#### Modelling

All experiments were performed twice on two different batches. The cell load data of probiotic strains were modelled according to the Gompertz equation modified by Zwietering et al. (1990):

(1) $$y{\rm{ }} = {\rm{ }}k + A*{\rm{exp}}\left\{ {-{\rm{exp}}\left[ {\left({{{\rm{\mu }}_{{\rm{max}}}}*e/A} \right)*\left({ABF-{\rm{time}}} \right) + 1} \right]} \right\}$$

where *y* is assumed as the biofilm cells population (Log (CFU cm^−2^)), *k* as the initial biofilm count equivalent to zero, *A* is the bacterial load attained at the stationary phase (Log (CFU cm^−2^)), μ_max_ as the maximal adhesion rate (Δ Log (CFU cm^−2^ day^−1^), *ABF* as the aptitude to BF, i.e., the time necessary to start adhesion on the surface (day) and *t* is the time (day).

### Phase II: evaluation of the effects of pH, temperature, cellular growth phase, agitation, and presence of surfactants on probiotic biofilm formation

#### Samples preparation

In order to evaluate the effects of pH, temperature, cellular growth phase, agitation and presence of surfactants on BF by *Bifidobacterium infantis* DSM20088 and *Lactobacillus reuteri* DSM20016, two 2^k-p^ Fractional Factorial Designs were developed (Box, Hunter & Hunter, 2005). The coded values and the combinations tested are reported in Table 2.

**Table 2:** Coded values and combinations tested in the 2k-p fractional factorial designs.

Coded values	pH	Agitation	Surfactants	Cellular growth phase	Temperature (°C)
−1	4.5	Yes	Yes	Exponential	15
1	8.5	No	No	Stationary	45
**Combinations**					
A	4.5	No	Yes	Exponential	45
B	8.5	No	No	Stationary	45
C	8.5	No	No	Exponential	15
D	8.5	Yes	Yes	Stationary	45
E	4.5	Yes	No	Exponential	45
F	4.5	No	Yes	Stationary	15
G	8.5	Yes	Yes	Exponential	15
H	4.5	Yes	No	Stationary	15

**Note:**

Coded values and combinations tested in the 2k-p fractional factorial designs about the effects of pH, temperature, cellular growth phase, agitation and presence of surfactants on biofilm formation by *B. infantis* DSM20088 and *L. reuteri* DSM20016.

As model surface, polycarbonate resin was chosen. As surfactant, sodium dodecyl sulphate (SDS; Sigma-Aldrich, Milan, Italy) was used at a concentration of 2% (w V^−1^). To allow BF, chips (2.5 × 5.0 × 0.05 cm) were placed vertically into sterile polypropylene containers (50 mL) filled with 45 mL of MRS medium (Oxoid, Milan, Italy) (one chip into one container). The chips were totally covered by the medium (no air presence) and each container was closed with a lid to avoid any gas exchange from inside to outside.

Incubation temperature, pH, presence/absence of surfactant, presence/absence of agitation were modulated according to Table 2. Samples were inoculated at 10^2^ CFU mL^−1^ by using an 18 h preculture (exponential growth phase) or a 30 h preculture (stationary growth phase) according to the design and then incubated for three days. Agitation was performed placing the samples on an orbital shaker (0–150 rpm): the maximum value of agitation was chosen after some preliminary experiments showing that higher values caused the formation of vortices into the jars making this operation unstable. Biofilm cells were enumerated at 1, 2, and 3 days after inoculum, as previously described.

#### Modelling

All experiments were performed twice on two different batches. The sessile cell loads after 1, 2, and 3 days were used as input values for a black-box model analysis; the statistical analysis was performed through the option Design of Experiments (DoE) of the software Statistica for Windows (StatSoft, Tulsa, OK, USA).

The polynomial equation was in the following form:

(2) $$y = {{{\beta }}_0} + {\sum\limits_{j = {\rm{1}}}^n {{{\rm{\beta }}_j} \cdot {X_j}} } + {\sum\limits_{j = {\rm{1}}}^n {\sum\limits_{k = j + {\rm{1}}}^n {{{\rm{\beta }}_{jk}} \cdot {X_j} \cdot {X_k}} } } + {\sum\limits_{j = {\rm{1}}}^n {{{\rm{\beta }}_{jj}} \cdot {X_{{j^{\rm{2}}}}}} }$$

where: ${\sum\nolimits_{j = {\rm{1}}}^n {{{\rm{\beta }}_j} \cdot {X_j}} }$ is the individual effect of each factor (independent variable); ${\sum\nolimits_{j = {\rm{1}}}^n {\sum\nolimits_{k = j + {\rm{1}}}^n {{{\rm{\beta }}_{jk}} \cdot {X_j} \cdot {X_k}} } }$ indicates the interactions among the variables; the term ${\sum\nolimits_{j = {\rm{1}}}^n {{{\rm{\beta }}_{jj}} \cdot {X_{{j^{\rm{2}}}}}} }$ takes into account a possible non-linear/quadratic effect of some factors; *y* is the dependent variable (sessile cell count) (Van Boekel & Zwietering, 2007).

The significance of the polynomial equation was evaluated through the adjusted regression coefficient, as well as with Fisher test and standard error of the model. In addition, the statistical weight of each term was pointed out through the standardized effects associated with each individual, quadratic and interactive factors of the equations. These standardized effects were evaluated as the ratio of the mathematical coefficient of each term of the equation vs. the respective standard error.

### Phase III: study of the effects of pH, temperature and surfactants concentration on probiotic biofilm formation testing both an anionic surfactant and a non-ionic surfactant

#### Samples preparation

In order to study the effects of pH, temperature and surfactants concentration on BF by *B. infantis* DSM20088 and *L. reuteri* DSM20016, two 5 levels-3 variables Central Composite Designs (CCDs) were developed (Box, Hunter & Hunter, 2005; Bevilacqua, Corbo & Sinigaglia, 2010). For each strain, two surfactants were tested: an anionic surfactant (SDS; Sigma-Aldrich, Milan, Italy) and a non-ionic surfactant (Polysorbate 80; Sigma-Aldrich, Milan, Italy). The combinations tested are reported in Table 3.

**Table 3:** Levels and combinations tested in the 5 levels-3 variables central composite designs.

Levels	pH	Temperature (°C)	Surfactants (%)
−α (−2)	4	10	0
−1	5	20	0.5
0	6	30	1
+1	7	40	1.5
+α (+2)	8	50	2
**Combinations**			
1	7	40	1.5
2	7	40	0.5
3	7	20	1.5
4	7	20	0.5
5	5	40	1.5
6	5	40	0.5
7	5	20	1.5
8	5	20	0.5
9	6	30	1
10	6	30	0
11	6	30	2
12	6	10	1
13	6	50	1
14	4	30	1
15	8	30	1
16	6	30	0
17	6	30	0

**Note:**

Levels and combinations tested in the 5 levels-3 variables Central Composite Designs about the effects of pH, temperature and surfactants concentration on biofilm formation by *B. infantis* DSM20088 and *L. reuteri* DSM20016.

To allow BF, polycarbonate sterile chips were placed vertically into jars containing 45 mL of MRS medium (Oxoid, Milan, Italy). Incubation temperature, pH medium and surfactant concentration were modulated according to the designs. Samples were inoculated at 10^2^ CFU mL^−1^ by using a 30 h preculture (stationary growth phase) and then incubated for three days without agitation. Biofilm cells were enumerated at 3 days after inoculum, as previously described. Modelling was performed as described above. All experiments were performed twice on two different batches.

### Phase IV: effect of probiotic biofilms on *L. monocytogenes* growth

#### Experiment

As pathogen target was chosen a strain of *L. monocytogenes* from the collection of the Laboratory of Predictive Microbiology (Department of the Science of Agriculture, Food and Environment, University of Foggia); the organism was transferred to fresh Nutrient Agar (NA; Oxoid, Milan, Italy) periodically to maintain viability and, prior to use, it was activated by two successive 24 h transfers of cells in Nutrient broth (NB; Oxoid, Milan, Italy) at 37 °C. Inocula for experiments were prepared by centrifugation of the 24 h microbial cultures at 3,000 *g* for 15 min at 4 °C. Probiotic inocula were prepared following the procedure described above: the pellets obtained after centrifugation were resuspended in sterile isotonic solution temperate at 4 °C and serial dilutions were made with physiological solution (0.9% NaCl) to obtain approximately 10^3^ CFU mL^−1^ for each microorganism.

The surface used to get the biofilm attached was polycarbonate resin. To promote BF, polycarbonate sterile chips were placed vertically into jars containing 45 mL of MRS medium (Oxoid, Milan, Italy), inoculated with each probiotic strain (10^2^ CFU mL^−1^) and incubated at 30 °C. After three days, chips with biofilm were aseptically removed from the medium, rinsed with sterile distilled water to remove the unattached cells and transferred into test tubes containing fresh sterile NB (45 mL). Specifically, two samples were prepared: ACTIVE (ACT) samples, with probiotic biofilm (one formed by *B. infantis* and one formed by *L. reuteri*); CONTROL (CNT) samples, without probiotic biofilm. All tubes were inoculated with *L. monocytogenes* (10^2^ CFU mL^−1^) and incubated at 37 °C for two days. The pathogen cell load was determined after 0, 3, 6, 24, 30 and 48 h using Listeria selective agar base (Oxoid, Milan, Italy) plus Listeria selective supplement-Oxoid formulation, incubated at 37 °C for 48 h.

All experiments were performed twice on two different batches. The cell load data were modelled according to the Gompertz equation modified by Zwietering et al. (1990). The results were analysed through one-way ANOVA and Tukey’s test as the post hoc comparison test (*P* < 0.05).

## Results

**Figure 1:**
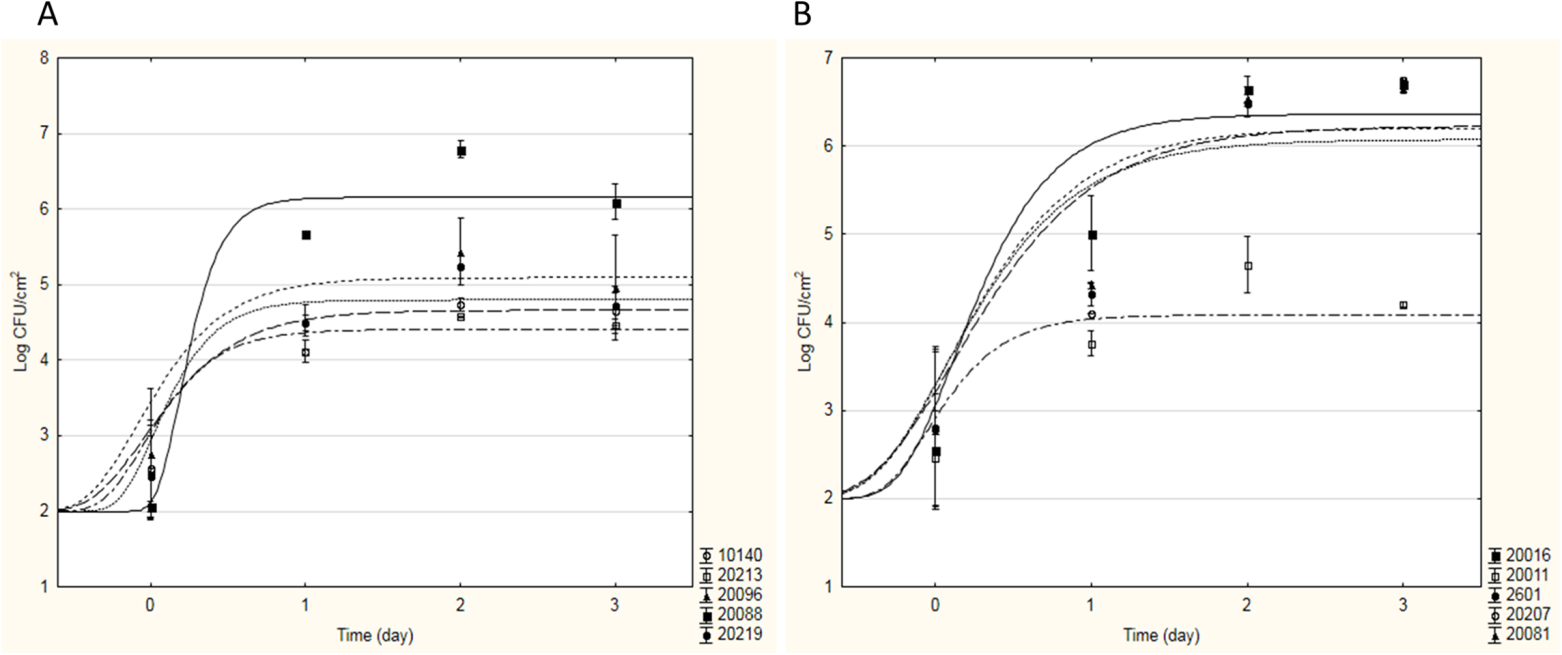
Sigmoidal curves of cell adhesion of bifidobacteria (A) and lactobacilli (B) strains on stainless steel. The curves represent the best fitting Gompertz equation to the experimental data obtained.

During Phase I, the adhesion capacity of 15 strains with probiotic potential (see Table 1) was measured in model systems and optimal growth conditions to evaluate their tendency to form biofilm on stainless steel, glass and polycarbonate surfaces. As an example, Fig. 1 shows the sigmoidal curves of cell adhesion of bifidobacteria (a) and lactobacilli (b) strains on stainless steel; they represent the best fitting Gompertz equation to the experimental data obtained.

As can be seen from Fig. 1A, for all bifidobacteria, a good adhesion on stainless steel was recovered: all tested strains showed a similar behaviour, in terms of aptitude to BF (*ABF*, i.e. time necessary to start adhesion), maximal adhesion rate and sessile cellular load reached in the stationary phase, with the exception of *B. infantis* DSM20088. In fact, *B. animalis* DSM10140, *B. breve* DSM20213, *B. subtile* DSM20096, and *B. longum* DSM20219 started to adhere on stainless steel in a very short time (about 3 h after inoculation), reaching a maximum sessile cellular load of 4.51–5.20 Log CFU cm^−2^ between the first and the second incubation day. On the contrary, *B. infantis* DSM20088 was characterized by a longer *ABF* (13 h) and a greater adhesion rate; thus, even if it started to adhere later than the other bifidobacteria, this probiotic strain was able to reach a higher sessile cellular load (over 6 Log CFU cm^−2^) in a shorter time (<24 h). For this strain, high cellular loads in 24 h were also recovered for the adhesion experiments on glass and polycarbonate (about 5.13 Log CFU cm^−2^ and 4.42–5.20 Log CFU cm^−2^, respectively). About lactobacilli, each strain tested showed a good adhesion to the surfaces tested; as an example, Fig. 1B shows how four strains showed similar values of *ABF* and maximal adhesion rate, starting to form biofilm after few hours (2–3 h) and reaching high cellular loads in 24 h (6.22–6.49 Log CFU cm^−2^ on stainless steel and 5.71–6.32 Log CFU cm^−2^ on glass and polycarbonate). The only exception to this trend was *Lactobacillus casei* DSM20011; this strain adhered to the tested surfaces less than the other strains, observing a maximum sessile cellular load about 3.65–5.00 Log CFU cm^−2^.

All the yeast strains showed a minor capability to form biofilm than the other targets tested; particularly, no adhesion was observed for *Kluyveromyces lactis* ATCC8585.

To individuate the best probiotic strains able to adhere to the tested surfaces and form biofilm, Table 4 resumes the maximum sessile cellular loads reached in the stationary phase by each studied microorganism. These results allowed to identify in *B. infantis* DSM20088 the probiotic *Bifidobacterium* able to ensure the greatest adhesion to the surfaces tested; regarding lactobacilli, since all the studied strains showed similar results, *L. reuteri* DSM20016 was chosen, being considered an emergent probiotic (Abrahamsson et al., 2007; Saunders et al., 2007; Savino et al., 2007). Thus, in Phase II, the attention was focused only on these two strains; as model surface, only polycarbonate resin was chosen.

**Table 4:** Maximum sessile cellular loads (Log CFU cm^−2^) reached in the stationary phase by each studied microorganism.

Strains	Maximum sessile cellular load (Log CFU cm^−2^)		
Bifidobacteria	Stainless steel	Glass	Polycarbonate
*B. animalis* DSM10140	4.69 ± 0.08^A^	4.66 ± 0.22^A^	3.92 ± 0.33^A^
*B. subtilis* DSM20096	5.20 ± 0.24^A,B^	4.62 ± 0.10^A^	5.09 ± 0.02^B^
*B. infantis* DSM20088	6.44 ± 0.20^B^	5.13 ± 0.04^B^	4.81 ± 0.39^B^
*B. longum* DSM20219	4.97 ± 0.16^A^	4.41 ± 0,17^A^	5.31 ± 0.15^B^
*B. breve* DSM20213	4.51 ± 0.07^A^	4.81 ± 0.08^A^	4.07 ± 0.05^A^
**Lactobacilli**			
*L. plantarum* DSM2601	6.49 ± 0.07^A^	5.71 ± 0.13^A^	5.76 ± 0.23^A^
*L. casei* DSM20011	4.38 ± 0.15^B^	3.65 ± 0.11^B^	5.21 ± 0.16^B^
*L.delbrueckii* DSM20081	6.22 ± 0.311^A^	5.88 ± 0.35^A^	4.97 ± 0.21^B^
*L. paracasei* DSM20207	6.44 ± 0.06^A^	6.11 ± 0.29^A^	5.49 ± 0.17^A^
*L. reuteri* DSM20016	6.36 ± 0.23^A^	5.85 ± 0.23^A^	6.32 ± 0.20^C^
**Yeasts**			
*Kluyveromyces lactis* ATCC8585	No adhesion	No adhesion	No adhesion
*S.cerevisiae boulardii* ATCCMYA-796	2.94 ± 0.15^A^	2.99 ± 0.12^A^	2.96 ± 0.16^A^
S.cerevisiae W21*	3.16 ± 0.12^A^	3.24 ± 0.12^A^	3.26 ± 0.41^A^
S.cerevisiae W40*	4.02 ± 0.46^A,B^	4.59 ± 0.53^A,B^	4.08 ± 0.12^A,B^
S.cerevisiae W45*	3.03 ± 0.21^A^	3.20 ± 0.36^A^	3.86 ± 0.21^B^

**Notes:**

A, B, values in the same columns with different letters are significantly different (one-way ANOVA and Tukey’s test) (*P* < 0.05).

* Autochthonous yeasts isolated from wine (Petruzzi et al., 2014).

The effects of pH, temperature, cellular growth phase, agitation and presence of surfactants on BF by the selected probiotics were evaluated through two fractional factorial designs. This methodology assesses the most relevant variable acting on BF by using the maximum sessile cellular loads recovered for each strain (after 1 day) as input data the to run a black-box model through a DoE approach; sessile cell load was the output of this model (dependent variable), whilst the factors of the design were used as independent variables. The main result of this approach was a set of standardized effects, evaluated as the ratio of the mathematical coefficient of each factor and its standard error. These calculated standardized effects showed how each studied factor affected the output (in a positive or in a negative way) and if it was significant or not (Table 5); for both strains, the only factors playing a role were pH, temperature and presence of surfactant. Namely, in both cases pH and presence of surfactant exerted a negative effect on the adhesion capacity (when they increased, adhesion decreased), whereas the temperature had a positive effect (its increase caused greater adhesion). Not having a statistically significant effect, the effect of cellular growth phase on BF was not been further investigated; in the range tested (from 0 to 150 rpm), the effect of agitation was also not significant and consequently no longer studied.

**Table 5:** Standardized effects (evaluated as the ratio of the mathematical coefficient of each factor and its standard error).

	Time (day)		
	1	2	3
***B. infantis* DSM20088**			
Intercept	1.115	1.119	1.355
pH	−2.229	−2.219	−2.730
Agitation	ns^a^	ns	ns
Presence of surfactants	−2.229	−2.219	−2.730
Cellular growth phase	ns	ns	ns
Temperature	2.229	2.219	2.730
MS^b^	0.089	0.096	0.011
*R*^2^_adj_^c^	0.970	0.968	0.998
			
***L. reuteri* DSM20016**			
Intercept	1.139	1.312	1.380
pH	−2.289	−2.638	−2.788
Agitation	ns	ns	ns
Presence of surfactants	−2.289	−2.638	−2.788
Cellular growth phase	ns	ns	ns
Temperature	2.289	2.638	2.788
MS	0.011	0.023	0.004
*R*^2^_adj_	0.995	0.990	0.997

**Notes:**

Standardized effects (evaluated as the ratio of the mathematical coefficient of each factor and its standard error) of pH, temperature, cellular growth phase, agitation and presence of surfactants on biofilm formation by the selected probiotics (*B. infantis* DSM20088 and *L. reuteri* DSM20016).

a Not significant;

b Mean square residual;

c *R*^2^ adjusted regression coefficient.

In Phase III, in order to evaluate the effects of pH, temperature and surfactant concentration on BF by *B. infantis* DSM20088 and *L. reuteri* DSM20016, two 5 levels-3 variables CCDs were developed (Bevilacqua, Corbo & Sinigaglia, 2010). For each strain, Table 6 shows the linear and the quadratic terms for each factor of the designs performed using both an anionic surfactant (SDS) and a non-ionic surfactant (Polysorbate 80, PS80).

**Table 6:** Linear, interactive and quadratic terms for the effects of pH, temperature and surfactants concentration on biofilm formation by *B. infantis* DSM20088 and *L. reuteri* DSM20016.

	Sodium dodecyl sulphate (SDS)		Polysorbate 80 (PS80)	
	*B. infantis*	*L. reuteri*	*B. infantis*	*L. reuteri*
**Linear terms**				
[pH]	ns^a^	3.090	−2.289	−3.339
[temperature]	3.259	ns	7.622	8.321
[surfactant]	−2.880	−2.420	ns	ns
				
**Interactive terms**				
[pH]*[temperature]	ns	ns	ns	ns
[pH]*[surfactant]	ns	ns	ns	ns
[surfactant]*[temperature]	ns	ns	ns	ns
				
**Quadratic terms**				
[pH]^2^	ns	ns	ns	ns
[temperature]^2^	−2.435	ns	−7.228	−8.499
[surfactant]^2^	ns	ns	ns	ns
				
*R*^b^	0.902	0.977	0.998	0.885
SE^c^	1.008	1.022	0.977	1.039

**Note:**

Both an anionic surfactant (SDS) and a non-ionic surfactant (PS80) were used.

a Not significant;

b R, regression coefficient;

c SE, standard error.

About the use of SDS, the results were different for *B. infantis* DSM20088 and *L. reuteri* DSM20016; concerning the *Bifidobacterium*, temperature played a positive role as linear and quadratic term on BF. The term of surfactant concentration was negative as linear effect, that is sessile cell load decreased when surfactant amount increased. pH was not significant.

Concerning *L. reuteri* DSM20016, pH acted as a positive term, that is an increase in pH exerted a detrimental effect on BF. The effect of surfactant was similar to that reported for *B. infantis* DSM20088 with a negative linear term. Finally, temperature was not significant.

Two other outputs of this approach were the polynomial equations for each time, useful to predict BF (sessile cellular load reached after 3 days) in a wide range of pH (4–8), surfactant concentrations (0–2%) and temperature (10–50 °C). As an example, the equations for *B. infantis* DSM20088 (BFB) and *L. reuteri* DSM20016 (BFL) are reported in the following; in the brackets, there are the regression coefficient (R) and the standard error (SE):

(3) $${\rm{BFB\;(Log\;CFU\;c}}{{\rm{m}}^ - }^{\rm{2}}{\rm{)\; =\; }}0.185\;{\rm{ }}\left[ {{\rm{temperature}}} \right]\;-1.745\;\left[ {{\rm{surfactant}}} \right]\;-0.008\;{\left[ {{\rm{temperature}}} \right]^2}\;\left[ {{\rm{R,\;0}}{\rm{.902;\;SE,\;1}}{\rm{.008}}} \right]$$

(4) $${\rm{BFL\; (Log\;CFU\;c}}{{\rm{m}}^-}^{\rm{2}}{\rm{) }} = \;0.560\;\left[ {pH} \right]-5.665\;\left[ {{\rm{surfactant}}} \right]\quad\left[ {{\rm{R,\;0}}{\rm{.977;\;SE,\;1}}{\rm{.022}}} \right]$$

**Figure 2:**
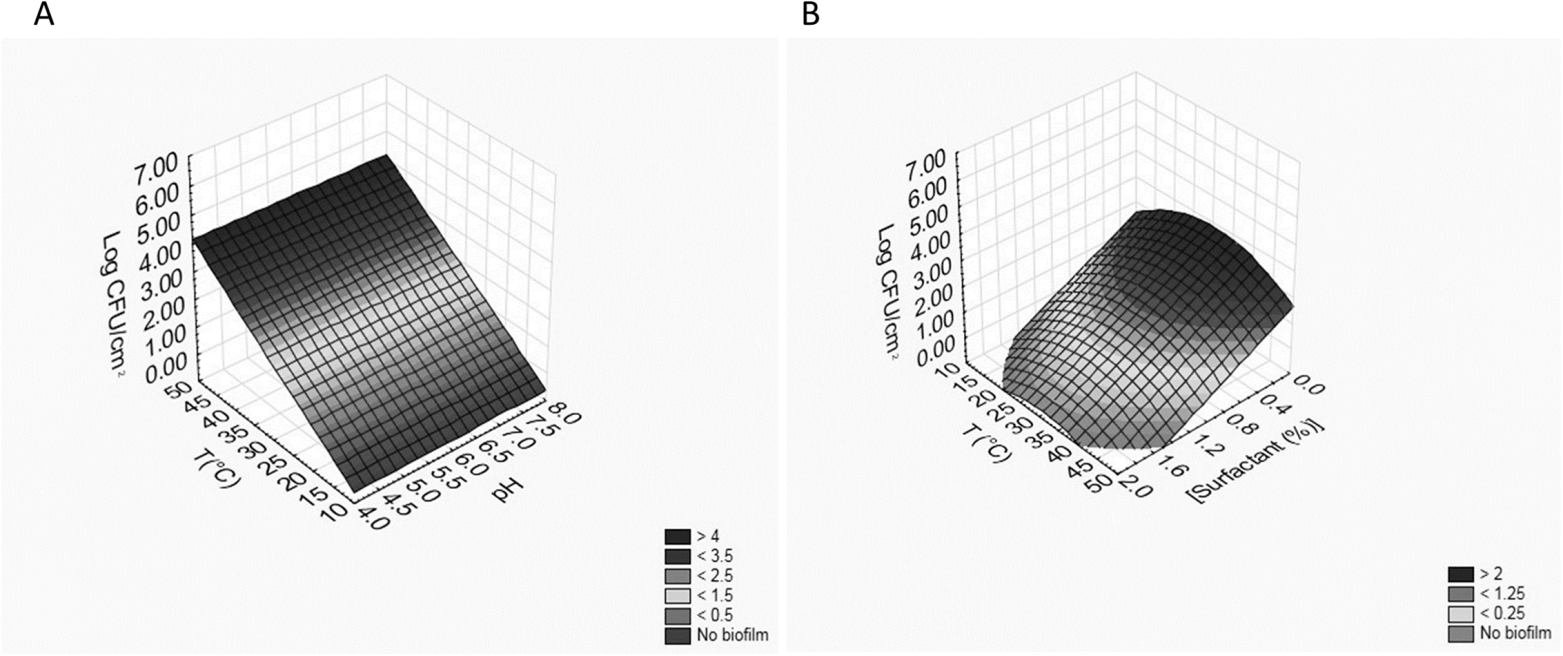
Effects of [pH]/[temperature] (A) and [surfactant]/[temperature] (B) on biofilm formation by *B. infantis* DSM20088.

These equations can be used to build 3D plots, showing the effects of the interaction of two variables on BF. As an example, Fig. 2 shows the effects of [pH]/[temperature] (a) and [surfactant]/[temperature] (b) on BF by *B. infantis* DSM20088.

Regarding the use of the non-ionic surfactant, the results obtained for *B. infantis* DSM20088 and *L. reuteri* DSM20016 were similar (Table 6); the terms of temperature were positive as linear effects, that is BF increased when temperature increased; however, the existence of a negative quadratic term pinpointed that the correlation was not strictly linear. The terms of pH were negative as linear effect, that is sessile cell load decreased when pH increased. The surfactant concentration, if maintained below 2%, was not significant. The equations for *B. infantis* DSM20088 (BFB) and *L. reuteri* DSM20016 (BFL) obtained during the use of the non-ionic surfactant are reported in the following; in the brackets, there are the regression coefficient (R) and the standard error (SE):

(5) $$\matrix{{{\rm{BFB}}\,{\rm{(Log}}\,{\rm{CFU}}\,{\rm{c}}{{\rm{m}}^ - }^{\rm{2}}{\rm{)}}} \hfill & = \hfill & { - 0.403\,\left[ {pH} \right]\, + \,0.599\,\left[ {{\rm{temperature}}} \right]-\,0.015\,{{\left[ {{\rm{temperature}}} \right]}^{\rm{2}}}\,} \hfill \cr {} \hfill & {} \hfill & {\,\,\,\,\left[ {{\rm{R,0}}{\rm{.998;SE,0}}{\rm{.885}}} \right]} \hfill\cr } $$

(6) $$\matrix{{{\rm{BFL (Log CFU c}}{{\rm{m}}^ - }^{\rm{2}}{\rm{)}}} \hfill & = \hfill & { - 0.660\,\left[ {pH} \right]\, + \,0.722\,\left[ {{\rm{temperature}}} \right]{\rm{-- 0}}{\rm{.024}}{{\left[ {{\rm{temperature}}} \right]}^{\rm{2}}}} \hfill \cr {} \hfill & {} \hfill & {\,\,\,\left[ {{\rm{R, 0}}{\rm{.979; SE, 1}}{\rm{.039}}} \right]\,\,} \hfill\cr} $$

**Figure 3:**
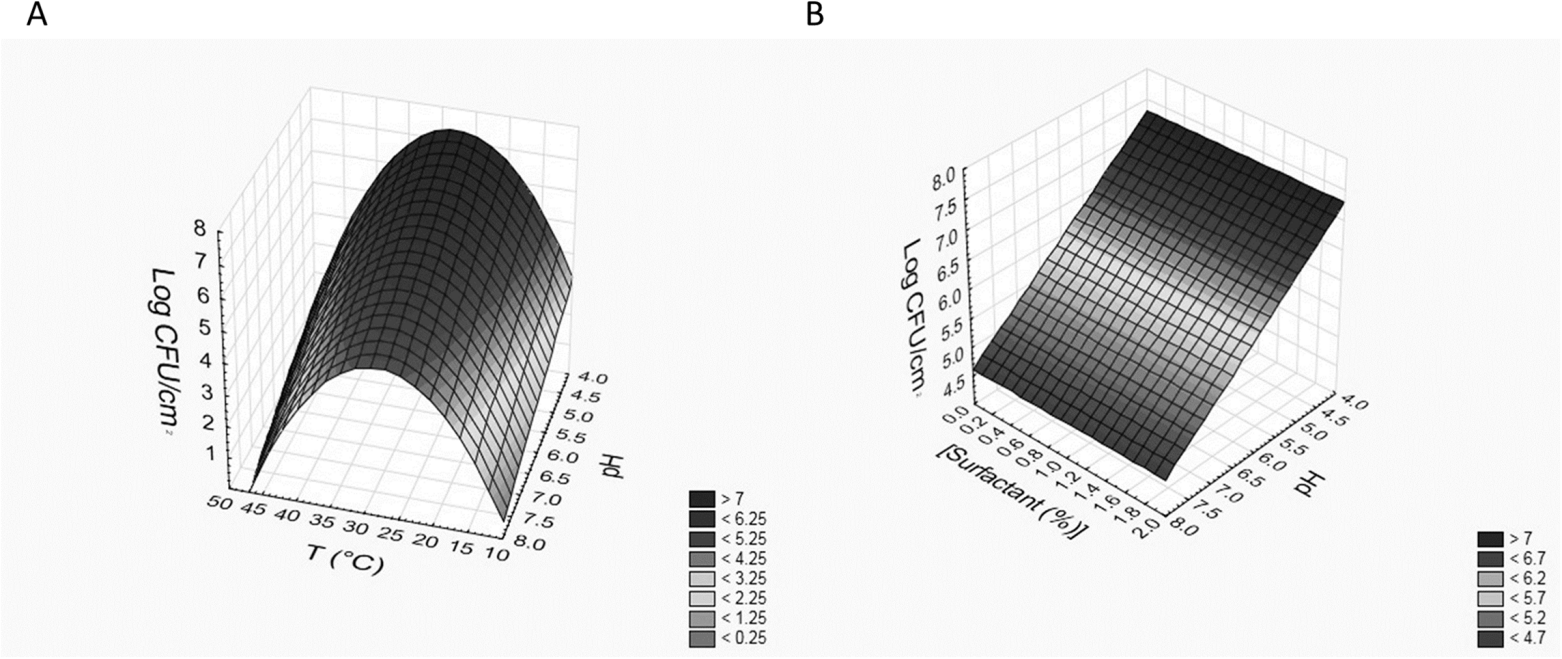
Effects of [pH]/[temperature] (A) and [surfactant concentration]/[pH] (B) on biofilm formation by *L. reuteri* DSM20016.

Also in this case, these equations can be used to build 3D plots, showing the effects of the interaction of two variables on BF. As an example, Figs. 3A and 3B shows the effects of [pH]/[temperature] (a) and [surfactant concentration]/[pH] (b) on BF by *L. reuteri* DSM20016.

To point out whether biofilm pre-formed by probiotics on polycarbonate surfaces could delay the growth of *L. monocytogenes*, this pathogen was inoculated (about 2 Log CFU mL^−1^) in the presence of chips with 3 days biofilms (sessile cellular load of 6.25–6.50 Log CFU cm^−2^). *L. monocytogenes* was able to proliferate in all samples (Table 7); however, the maximum cell load attained at the stationary phase (A) was always lower in ACT samples, recording values of 7.04 (± 0.06) and 7.28 (± 0.11) Log CFU mL^−1^ in presence of *B. infantis* and *L. reuteri* biofilms, respectively, against about 8 Log CFU mL^−1^ observed in the CNT samples. An increase in the λ phase was also recorded, 1.07 and 0.16 h in ACT samples against 0.09 h in CNT.

**Table 7:** Kinetic parameters calculated by fitting Gompertz equation to the experimental data by *L. monocytogenes* during its growth with (ACT) or without (CNT) probiotic biofilms.

	A	μmax	λ
	[LogCFU mL^−1^]	[ΔLog(CFU mL^−1^) h^−1^]	[h]
ACT *B. infantis*	7.04 ± 0.06^A^	0.79 ± 0.03^A^	1.07 ± 0.15^A^
ACT *L. reuteri*	7.28 ± 0.11^A^	0.70 ± 0.02^A^	0.16 ± 0.02^B^
CNT	7.98 ± 0.10^B^	0.84 ± 0.04^A^	0.09 ± 0.03^C^

**Notes:**

A is the maximum bacterial load attained at the stationary phase, μ_max_ is the maximal specific growth rate, λ is the lag time. A, B, C, values in the same columns with different letters are significantly different (one-way ANOVA and Tukey’s test) (*P* < 0.05).

## Discussion

The potential application of probiotics is continuously widening, with new evidences supporting their effect on the prevention of growth of several pathogenic and spoilage bacteria. Considering the increasingly widespread ability of pathogens and/or spoilage bacteria to generate persistent biofilm-related infections or contaminations, an even more attractive proposal is to use probiotics to prevent or counteract biofilm development. Some recent in vitro investigations have suggested a potential role of probiotic *Lactobacilli* and *Bifidobacteria* in controlling BF by replacing resident biofilm-growing pathogens with a non-pathogenic bacteriocin-producing variant (Aoudiaa et al., 2016; Jones & Versalovic, 2009; Zakaria, 2013). Despite this, biofilms developed by probiotic bacteria have so far been poorly investigated (Lebeer et al., 2007; Muscariello et al., 2013) as compared with the extensive studies performed on the BF of several microbial pathogens. Consequently, a study on the formation of probiotic biofilms and on the factors affecting this process may be useful for innovative biomedical, industrial, and food applications. During a first step, our research focused on the measurement of the adhesion capacity of 15 strains with probiotic potential to evaluate their tendency to form biofilm on different surfaces (stainless steel, glass and polycarbonate) and to individuate the probiotic strains more inclined to form biofilms. Among the tested strains, *B. infantis* DSM20088 and *L. reuteri* DSM20016 were individuated as the probiotics able to ensure the greatest adhesion (over 6 Log CFU cm^−2^) to the surfaces tested in a very short time (<24 h). Intriguingly, despite their ability to adhere to mucus and epithelial cells, biofilm development of bifidobacteria remains poorly investigated. To investigate the cariogenic potential of bifidobacteria, some authors (Valdez et al., 2016) tested *B. lactis*, *B. longum*, *B. animalis*, *B. dentium*, *Lactobacillus acidophilus*, *Lactobacillus casei*, *Actinomyces israelii*, *Streptococcus sobrinus*, and *Streptococcus mutans* for acidogenicity, aciduricity, and capacity to form biofilm; this tendency was determined for each species either alone or associated streptococci. In their study (Valdez et al., 2016), bifidobacteria and lactobacilli had lower ability to form biofilm as single-species compared with dual- and multi-species biofilms. On the other hand, multi-species growth of *B. longum*, *B. animalis*, *Lactobacillus casei*, and *Lactobacillus acidophilus* presented higher BF ability compared with their growth as dual-species. Similar studies had demonstrated that some bifidobacteria (*B. breve, B. longum, B. lactis, B. adolescentis, B. infantis*) exhibited low adhesion to hydroxyapatite discs (Haukioja et al., 2006; Nagaoka et al., 2008), but this ability was improved when the species had co-adhered with primary colonizers, such as *Actinomyces naeslundii*, *Veillonella parvula*, and *Fusobacterium nucleatum*. In this study, for all bifidobacteria, a good adhesion was recovered with *B. infantis* DSM20088 able to reach a high sessile cellular load in a very short time.

With respect to lactobacilli, each strain tested showed a good adhesion to the surfaces used reaching high cellular loads (6.22–6.49 Log CFU cm^−2^) after few hours (2–3 h); only for *Lactobacillus casei* DSM20011 a minor sessile cellular load was observed (about 3.65–5.00 Log CFU cm^−2^). In 2016 Aoudia et al. evaluated the ability of three *Lactobacillus* strains (one strain of *L. plantarum* and two strains of *L. fermentum* isolated from human feces or saliva) to form biofilms on polystyrene; BF was observed for all the *Lactobacillus* strains, but *L. fermentum* NA6 was able to form the most robust binding. Jones & Versalovic (2009) also observed a different adhesion on polystyrene by various isolates of *L. reuteri*, depending on the strain; this strain-dependence was confirmed for numerous strains of *Lactobacillus* genus (Lebeer et al., 2007; Martín et al., 2008).

All the yeast strains tested in this study showed a low capability to form biofilm. It is well recognized that yeast cells possess a remarkable capacity to adhere to abiotic surfaces, cells and tissues, but the major emphasis on yeasts adhesion properties remains about pathogenic ones such as *Candida albicans* and *Candida glabrata* and their ability to adhere to medical devices and form drug-resistant biofilms (Verstrepen & Klis, 2006). Despite BF is a desirable property of industrial *Saccharomyces cerevisiae* strains, this aspect remains poorly studied making difficult to compare the obtained results with others present in the literature.

Once individuated in *B. infantis* DSM20088 and *L. reuteri* DSM20016 the probiotic strains able to ensure the greatest adhesion to the surfaces tested, in Phase II, we focused on these two strains. Moreover, since both the probiotic strains adhered similarly to the tested surfaces and formed biofilm, in the following phases we choose to use only polycarbonate resin for its specific characteristics. Polycarbonate, in fact, is a strong, transparent, inert, tough material, easy to work with and it can undergo large plastic deformations without cracking or breaking making it valuable in prototyping applications (Kausar, 2017).

Several conditions are able to influence biofilm development including environmental variables (pH, nutrient availability, temperature, fluid dynamics), microbiological factors (Gram negative/positive, microbial shape, structure, species, growth phase, age, presence of flagella, pili, capsules or exopolymeric substances) and surface morphologies (chemistry, topography, physicochemistry) (Guðbjornsdottir, Einarsson & Thorkelsson, 2005). Among these factors, pH, temperature, medium composition and population characteristics of bacteria play an important role in the phenotypic change from planktonic cells to the sessile form, but their effects are not unique and may differ from species to species; for instance, it was demonstrated that maximum adhesion occurred at different pHs or temperatures depending on the tested strain. Also the physiological status of cells influences the hydrophobicity and the degree of bacterial adhesion; for example, it is recognized that spores possess higher hydrophobicity of their cell surfaces, thus adhering more quickly than vegetative cells to food-contact surfaces (Speranza & Corbo, 2017). Since adherence depends both on the strain and on environmental factors (Speranza & Corbo, 2017), we focused on the study of the effects of pH, temperature, cellular growth phase, agitation and presence of surfactants on BF by the selected probiotics. For both strains, the effects of cellular growth phase and agitation (until 150 rpm) on BF were not statistically significant, thus only the effects of pH, temperature and surfactant concentration were further investigated, using both an anionic surfactant (SDS) and a non-ionic surfactant (Polysorbate 80, PS80). Surfactants are generally employed to lower the surface tension of a surface as they are amphiphilic molecules, i.e., they have both hydrophilic and hydrophobic moieties. They are able to adhere easily on surface, thus inhibiting the adhesion by microorganisms (Toutain-Kidd et al., 2009). In general, surfactants can be cationic, anionic and non-ionic: the first (i.e. quaternary imidazolium compounds) are the most toxic, the second ones (i.e., SDS) are the most effective, whereas non-ionic surfactants generally have no antibacterial activities (Van Hamme, Singh & Ward, 2006). PS80 is a water soluble non-ionic surfactant which is very well tolerated (National Toxicology Program, 1992) and thus it is commonly used in foods, cosmetics, and pharmaceutical preparations. In this study, in tests using SDS, BF by both probiotics was influenced negatively by surfactant concentration, that is sessile cell load decreased when surfactant amount increased. On the other hand, when the surfactant used was PS80, its effect was not significant and both probiotics were able to reach high sessile cellular loads (over 6 Log CFU cm^−2^). Some studies have addressed the ability of surfactants to affect BF by other strains (namely pathogens) and have obtained different results. For example, it has been demonstrated that PS80 can inhibit the biofilms by *Pseudomonas aeruginosa* PA14 (Toutain-Kidd et al., 2009) and by *E. coli* O104:H4 (Sloup et al., 2016). Mireles, Toguchi & Harshey (2001) reported that SDS and PS80 (used at 0.25 g L^−1^) were effective at inhibiting BF by *E. coli*, *Salmonella enterica*, and *Proteus mirabilis* on catheters, but not BF by *P. aeruginosa*, reflecting the diversity in the nature and recalcitrance of biofilms produced. Other authors (Díaz De Rienzo et al., 2016) compared the effects of SDS with the ability of the rhamnolipids (biosurfactants from *P. aeruginosa*), in the presence and absence of caprylic acid and ascorbic acid, to disrupt bacterial biofilms: the SDS had a clear effect on *P. aeruginosa* ATCC 15442 biofilm disruption at 0.8 g L^−1^. SDS has also been shown to kill planktonic *Aggregatibacter actinomycetemcomitans* cells at a minimum inhibitory concentration (MIC) of 0.1 g/l (Díaz De Rienzo et al., 2016). Otzen (2002) suggested that SDS mediated biofilm detachment was a consequence of the denaturation of proteinaceous matrix adhesions.

Regarding the effects of pH and temperature, pH exerted a very bland effect, whereas it was possible to recover a greater tendency to adhesion when the temperature was about 30 °C. This more rapid transition from planktonic to sessile mode of life observed at 30–32 °C rather than at more suitable conditions (the optimal temperature for *Lactobacilli* and *Bifidobacteria* is 37 °C) is not a surprising result, even if the mechanisms behind increased BF at suboptimal temperatures are not known. Also in this case, such evidences were exclusively obtained in studies performed on pathogens; for example, in 2011 Speranza, Corbo & Sinigaglia observed a greater tendency to form biofilm by *Salmonella* sp. at 30–32 °C and neutral pH. Similar results were reported by Stepanovic et al. (2003) who proposed that the production of fimbriae explained an increased biofilm production of *Salmonella enterica* serovar Typhimurium at 30 °C. Research on *L. monocytogenes* has shown that temperature affects the bacterial hydrophilic surface properties (Chavant et al., 2002), with low temperatures increasing the cells hydrophilic properties and altering the bacteria’s ability to adhere to hydrophobic materials. It is widely accepted that each biofilm is different, due to the wide range of contributing factors (surface type, availability of nutrients and oxygen, microbial species, etc.) (Srey, Jahid & Ha, 2013); thus, every situation should be analyzed individually and specifically.

Finally, to test whether biofilm pre-formed by probiotics could delay the growth of pathogens, in the last phase of this research we conducted experiments evaluating their effect on *L. monocytogenes* growth chosen as target organism, due to its wide distribution in nature and importance both in food processing and medical environment. In fact, listeriosis is a severe infection associated with foods contaminated by *L. monocytogenes*, but also often implicated in nosocomial outbreaks. Some studies of listeriosis have also identified hospitalization as a risk factor; in hospitals, in fact, the target populations of this infection (pregnant women, newborn infants, immuno-compromised individuals on corticosteroids, patients with cancer and other chronic diseases and the elderly) are more present and the transmission can occur not only through contaminated foods, but also through person to person spread and direct contact with infectious material (Dalton et al., 2011).

In 2009 Guerrieri et al. observed that LAB biofilms were able to influence the survival and the growth of *L. monocytogenes* with differences among the strains: *L. plantarum* 35d was able to reduce the pathogen by 5.4 Log in the planktonic population and by 3.9 Log in the sessile population during 10 days of experimentation. Similar results were obtained by Speranza, Sinigaglia & Corbo (2009) who evaluated the possibility to consider non-starter LAB biofilms as a means to control the growth of *L. monocytogenes* in soft cheeses; their results demonstrated that biofilms were able to delay the growth of the pathogen by reducing the maximum cell load attained at the stationary phase (6 Log CFU g^−1^ in presence of biofilms against about 7 Log CFU g^−1^ observed in the control samples) and increasing the λ phase (2.87 against 0.78 days). These results confirm those obtained in the last phase of this research where the proposed probiotic biofilms were able to delay the growth of *L. monocytogenes.* Such effect was probably due to the release of different antimicrobial substances produced by the probiotic bacteria under the regulation of a quorum sensing mechanism when a threshold cell density was reached; this phenomenon occurs widely in LAB (especially for the bacteriocin production) in the presence of competitive microorganisms sensitive to their metabolites (Eijsink et al., 2002; Maldonado, Jiménez-Díaz & Ruiz-Barba, 2004).

Further investigations are warranted in order to explore whether our results can be reproduced and exploited in situ.

## Conclusion

This study is a valuable contribution to the study of probiotics’ ability to adhere to surfaces and form biofilms which allows to fix some key points:

1. Among the tested strains, *B. infantis* DSM20088 and *L. reuteri* DSM20016 were individuated as the probiotics able to ensure the greatest adhesion (over 6 Log CFU cm^−2^) to the surfaces tested in a very short time (<24 h);
2. All the yeasts with probiotic potential tested in this study showed less capability to form biofilm than bacteria;
3. For *B. infantis* DSM20088 and *L. reuteri* DSM20016, cellular growth phase and agitation of the medium (until 150 rpm) were factors not affecting BF;
4. Within the concentrations tested (0–2%), the non-ionic surfactant (PS80) was more delicate than anionic one (SDS). In fact, BF by both probiotics was negatively influenced by SDS concentration (sessile cell load decreased when surfactant amount increased), whereas PS80 had not significant effect and both probiotics were able to reach high sessile cellular loads (over 6 Log CFU cm^−2^);
5. pH variation exerted a very bland effect;
6. A greater tendency to adhesion was observed when the temperature was about 30 °C.
7. The results obtained in the last experimental phase suggest that these probiotic biofilms could be used as an efficient means to delay the growth of *L. monocytogenes*, even if this aspect requires further investigations.

Other studies are necessary to explore whether these bacteria are able to adhere on other surfaces (i.e. packaging materials, ceramic, plastic, paper, polymers, etc.) in order to develop new biotechnological solutions for industrial and medical applications.

## Supplemental Information

10.7717/peerj.4826/supp-1 Supplemental Information 1 Raw data. Each data point indicates the mean value of two or more repetitions.

10.7717/peerj.4826/supp-2 Supplemental Information 2 Extra dataset.

## Additional Information and Declarations

### Data Availability

The following information was supplied regarding data availability: The raw data obtained during experiments are provided in the Supplemental Files.
